# The value of Xpert MTB/RIF assay of urine samples in the early diagnosis of smear-negative urinary tuberculosis

**DOI:** 10.1186/s40001-022-00947-x

**Published:** 2022-12-20

**Authors:** Yachun Wang, Jiao Tan, Lei Lei, Yingying Yuan, Wenbo Li, Yue Zhao, Yali Wang, Xiaodong Niu, Zheng Li, Lukuan Wei, Yungang Han, Meijing Cheng, Xu Guo, Xue Han, Wei Wang

**Affiliations:** 1 Medical Laboratory, Henan Provincial Chest Hospital, Zhengzhou University, Zhengzhou, 450003 China; 2 Henan Provincial Key Laboratory of Tuberculosis Diagnostic Medicine, Zhengzhou, 450003 China; 3 Henan Provincial Infectious Diseases (Tuberculosis) Clinical Medical Research Center, Zhengzhou, 450003 China; 4 Department of Family Medicine, Henan Provincial Chest Hospital, Zhengzhou, 450003 China

**Keywords:** Smear-negative, Culture, Xpert MTB/RIF assay, TB-DNA, Urinary tract tuberculosis

## Abstract

**Background:**

According to reports, between 30 and 40 percent of extrapulmonary tuberculosis (EPTB) cases are caused by urinary tract tuberculosis (UTB). It is critical to identify UTB quickly since it frequently precedes delayed medical attention, which can have detrimental effects. This study examined the use of Xpert MTB/RIF, a PCR test that can detect MTB as well as resistance to an important drug, rifampicin (RIF), in UTB particularly, for the early identification of UTB.

**Methods:**

180 participants with clinically presumptive UTB whose urine samples were chosen for urine sediment smear, culture, Xpert MTB/RIF, and TB-DNA testing at Henan Chest Hospital between January 2019 and July 2022. Evaluation of test performance using Composite Reference Standards (CRSs). We studied and compared the positivity rate for various tests using the t-test. The effectiveness of smear, culture, Xpert MTB/RIF, and TB-DNA was assessed using McNemar test.

**Results:**

In this subject, a total of 108 participants were diagnosed with UTB, and the positivity rate was 67.1%. Compared with CRS, the positivity rate of Xpert MTB/RIF, smear, culture, and TB-DNA was 29.69% (19/64, *P* < 0.001), 7.56% (9/119, *P* < 0.1), 12.12% (4/33, *P* > 0.05), and 18.75% (6/32, *P* < 0.1), respectively. The sensitivity of Xpert MTB/RIF assay was significantly better than that of smear and culture tests (78.9% vs. 77.8%, *P* < 0.05; 78.9% vs. 75%, *P* < 0.05). Under CRS, the positivity rate for Xpert, culture, and TB-DNA was 31.6% (6/19, *P* < 0.1), 6.2% (1/16, *P* > 0.05), and 26.7% (4/15, *P* > 0.05) for TB-DNA, respectively, compared to smear negative. Xpert MTB/RIF assay specificity was significant for culture and TB-DNA (53.6% vs. 25%, *P* < 0.01; 53.6% vs. 38.9%, *P* < 0.05), and Xpert MTB/RIF assay FPV was significant for culture and TB-DNA (53.6% vs. 0%, *P* < 0.001; 53.6% vs. 0%, *P* < 0.001).

**Conclusion:**

Xpert MTB/RIF outperforms smear, cultures, and TB-DNA in detecting UTB, plus Xpert MTB/RIF is better suited for early diagnosis in smear-negative UTB.

## Background

The persistence of tuberculosis (TB) infection is incredibly troublesome and critical in general medical conditions worldwide. As indicated by the World Health Organization (WHO), TB is an irresistible infection brought about by *Mycobacterium tuberculosis* (MTB), which is a danger to human well-being, and its death rate has surpassed that of HIV/AIDS [1]. According to the Global TB Report 2022, there will be 10.6 million new cases of TB globally in 2021, with a cumulative decrease of 10% in TB incidence between 2015 and 2021; while China will have 780,000 new cases, ranking 3rd out of 30 high TB burden countries after Indonesia (969,000) and India (2.95 million). [2] TB can be divided into pulmonary tuberculosis (PTB) and extrapulmonary tuberculosis (EPTB), while the occurrence of EPTB is fundamentally an expanded pattern [3, 4]. Urinary tuberculosis (UTB) is a more serious kind of EPTB, the occurrence pace of which represents around 4% of TB, and its frequency rate in participants with EPTB is 30% to 40% [5]. The initial phase of UTB infection is often asymptomatic, which often leads to a delay in judgment. Its clinical side effects mostly appear in the late stage of the disease, for example, bladder contracture, contralateral hydronephrosis, and incomplete closure of the ureteral orifice, which later contributes to the annihilation of the urinary framework organ, further exemplifying the significance of early and rapid diagnosis. [6, 7]. Early determination and early treatment of UTB contamination is the way to control the spread of TB, so it means quite a bit to track down a sensible and compelling demonstrative technique to further develop the diagnosis rate of UTB.

At present, the determination of renal TB for the most part depends on imaging and etiological assessments. Imaging examinations of UTB, including B-ultrasound, Intravenous urography (IVU), and Computed tomography (CT), all of which are important in the diagnosis of UTB and help make judgments about the location, extent, nature, evolution, and effectiveness of treatment of the lesions. However, they do not change significantly in the early stages of UTB, so that the etiological assessment is vital in the early diagnosis of renal UTB.

Xpert® MTB/rifampicin (MTB/RIF) assay (Cepheid Inc., Sunnyvale, CA, USA) [8] is a PCR test that can detect MTB as well as resistant to an important drug, RIF [9]. However, the validity and role of the Xpert MTB/RIF assay for the diagnosis of urinary TB remain unclear, as most studies, including meta-analyses, have assessed the efficacy of combined EP samples rather than urine samples alone [10]. This study discusses the early diagnosis of renal TB and RIF resistance by Xpert MTB/RIF technology.

## Methods

### Study design and participants

This is a forthcoming report, for assessing the value of Xpert MTB/RIF assay in participants with smear-negative UTB. This study carried out in Henan provincial chest hospital from January 2018 to July 2022 in China. Participants in this study were aged 15 to 86 years with clinical or radiological features suggestive of UTB, such as participants with severe storage lower urinary tract symptoms (LUTS), hematuria, sterile pyuria, genital TB disorders, or participants with newly diagnosed TB at other sites where UTB was suspected. 19 participants who were HIV positive or had recently gotten anti-TB therapy for over one month or had recently gotten anti-TB therapy for over one month or had a background marked by a medical procedure to eliminate kidneys obliterated by TB were prohibited. Participants in this study were asked to provide urine samples for examination, including smear, culture, Xpert MTB/RIF, and TB-DNA testing. Given that M. tb culture is not ideal for the detection of EPTB, the Comprehensive Reference Standard (CRS) was also used as a reference standard in this study in the absence of a perfect gold standard, as recommended by relevant scholars.[11, 12]. In brief, this study divided the participants into two groups according to CRS: the UTB group and the Not-UTB group. The UTB group can include three more, i.e., the probable UTB group is participants with positive imaging, i.e., with a positive cystoscopy biopsy, or positive radiology; the probable UTB group is participants who influence anti-TB therapy; and the plausible UTB group is participants with clinical symptoms consistent with UTB. Not-UTB were participants without the above indicators and those who improved without anti-TB treatment [13, 14]. Participants were tested for smear, culture, Xpert MTB/RIF, and TB-DNA and the results were analyzed.

### Acid-fast Bacilli (AFB) smears

After centrifugation of each urine sample, 0.1–0.2 ml of the sample was taken for direct smear and stained with Ziehl–Neelsen anti-acid stain. These slides with samples were then microscopically examined with a light-emitting diode (LED) microscope. Finally, the slides were observed and counted under the microscope, the number of acid-fast bacilli greater than or equal to 3 per 100 visual fields was regarded as positive [15].

### Xpert MTB/RIF assay

Urine samples were mixed with Xpert MTB/RIF sample reagent at a ratio of 1:2, incubated for 15–30 min (room temperature); then 2 ml of Xpert MTB/RIF kit was added and loaded into the test system GeneXpert® Infinity-48 s. (Cepheid, Sunnyvale, CA). The system was then run and automatically produced results in the presence of MTB and RIF resistance after approximately 2 h. The results were interpreted according to the cycle threshold (Ct) value of the probe. When the Ct value of the internal control probe is less than or equal to 38, it is positive, otherwise, it is invalid (it indicates that the sample DNA extraction is unqualified or contains PCR inhibitors). The Ct value of at least 2 probes in the 5 probes is less than or equal to 38 to detect MTB, and MTB can be further semi-quantified according to the Ct value: if the Ct value is less than 16, it is high content, 16–22 is medium content, 22–28 is low content, and more than 28 is very low content. The basis for detecting RIF resistance is the difference between the early Ct value and the late Ct value of the MTB-specific molecular beacon, that is, Δct. The result interpretation standard set by the system: RIF resistance (Δct greater than or equal to 3.5) and RIF sensitivity (Δct less than 3.5). Since the number of termination cycles was 38 cycles when the Δct of the early probe was greater than 34.5 or the Δct of the late probe was greater than 38, the result of RIF resistance was indeterminate.

### MGIT 960 preparation and culture for MTB

Two mL of the centrifuged urine sample was taken, and the mixture of 2% N-acetyl-L-cysteine, sodium hydroxide, and sodium citrate to the digestion solution was added1–2 times, vortexed for 30 s, and let to stand at room temperature for 15 min. After finishing, phosphate buffer saline (PBS) (pH = 6.8) was added to 45 mL and centrifuged at 3000 × g for 15 min in a cryogenic centrifuge. After centrifugation, the supernatant is poured off, and after resuspension of the pellet by adding 0.8 mL of PBS, 0.8 mL of the sample sediment is added to the MGIT 960 liquid culture tube and incubated in the MGIT 960 machine for 42 days before a negative result will be determined. If the results of 42 days of culture were positive, they were stained with Ziehl–Neelsen and observed by fluorescence microscopy, as well as MTB antigen detection (Kaibili, Genesis, China). If acid-resistant bacilli were observed microscopically and the antigen was positive, the culture was determined to be positive. After testing for culture and antacid staining, no antacid bacilli were found, and the result was defined as culture negative. If the MGIT 960 tube remained negative for 42 days, this result was also defined as culture negative. All procedures were in accordance with the MGIT 960 instructions [16].

### MTB deoxyribonucleic acid (TB-DNA) PCR

After the urine was centrifuged, the supernatant was discarded, 4 times 4% NaOH was added to mix well, and it was liquefied at room temperature for more than 2 h. 500ul of the liquefied sample was taken and added to the nucleic acid extraction reagent. The next steps of extraction and amplification follow the instructions (Ex-DNA MTB, TIAN LONG, China).

### Statistical analysis

The measures used to quantify diagnostic significance in this study included sensitivity, specificity, false positivity value (FPV), false negativity value (FNV), and positivity rate. Sensitivity, specificity, FPV, and FNV were estimated 95% CI using bootstrap method and resampled 1000 times using the 2 × 2 table bootstrap method and the McNemar test was used to compare sensitivity. Unpaired t-tests were used to compare the number of positives and the total number in each experiment. Unpaired t-tests were performed using GraphPad Prism 7. All results are presented as the sample mean ± SD. A *p*-value of < 0.05 was considered statistically significant.

## Results

**Fig. 1:**
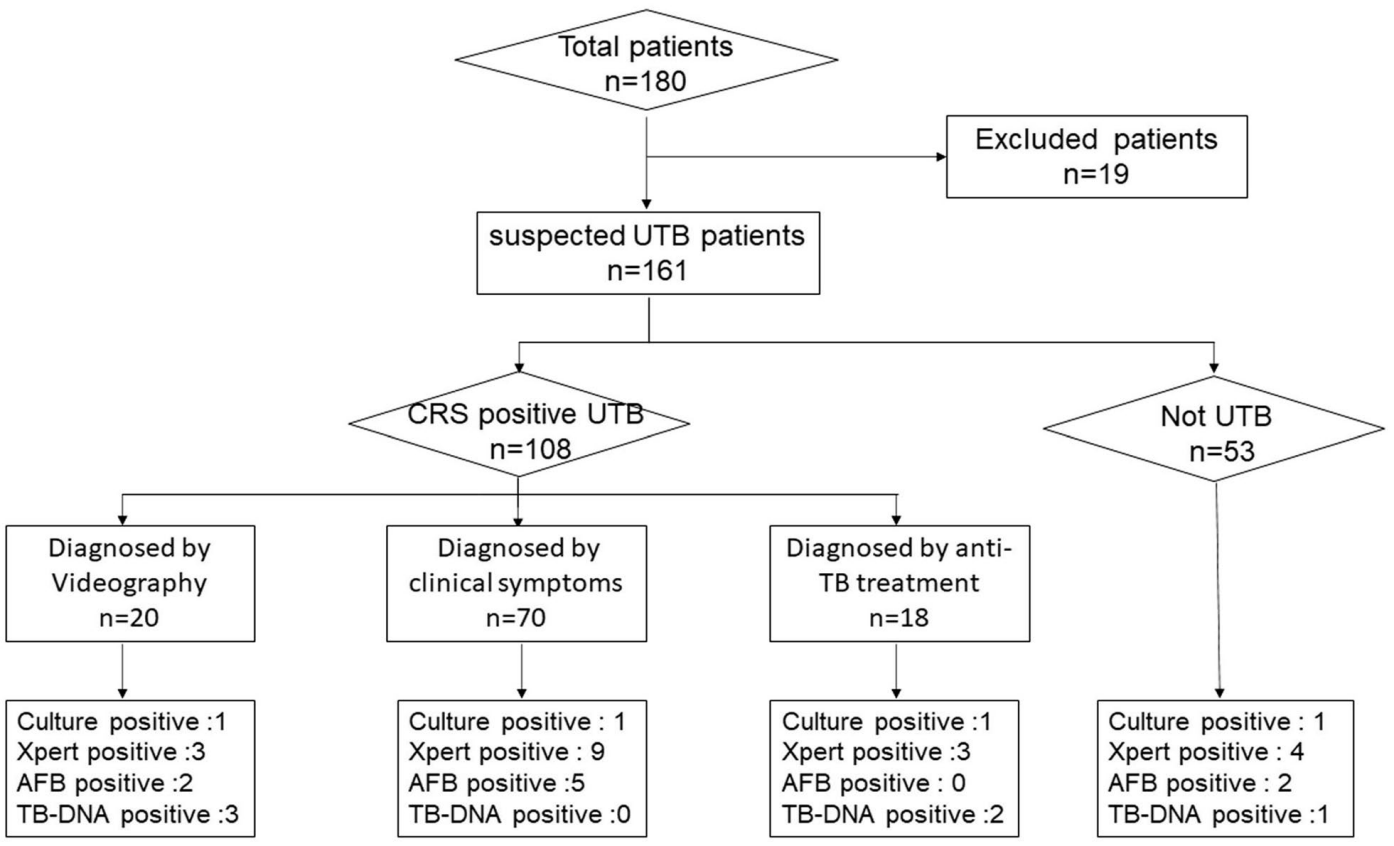
The flow of participant inclusion (all percentages report the proportion of 180 participants). *UTB* urinary tuberculosis *n* number *CRS* composite reference standard *AFB* Acid-Fast Bacilli smears *Xpert* Xpert MTB/RIF *culture* culture for MTB *TB-DNA Mycobacterium tuberculosis* deoxyribonucleic acid

In this study, a total of 180 urine samples were collected, of which 19 unqualified participant samples were excluded. Ultimately, a total of 161 samples have participated in this study. Among them, 108 were diagnosed UTB participants clinically (20 participants diagnosed based on imaging, 70 on clinical symptoms, and 18 were treated empirically with clinical anti-TB drugs), and 53 were Not-UTB participants. The process for selecting participants is shown in Fig. 1.

Of the 161 participants, 64 (39.8%, *P* > 0.05) were male with a mean age of (49.9 ± 17.7) years and 97 (60.2%, *P* > 0.05) were female with a mean age of (49.9 ± 13.1) years. The gender distribution was mostly female participants, and the age distribution was predominantly middle-aged participants, with 110 participants (68.3%, *P* < 0.05) older than 30 years and younger than 60. Basic information about the study participants is shown in Table 1.

**Table 1:** Distribution by gender and age

	CRS positive	CRS negative	Total	Relative share in (%)
Male	40	24	64	39.8
Female	68	29	97	60.2
Age < 30 years	14	1	15	9.3
30–60 years	71	39	110	68.3*
Age > 60 years	23	13	36	22.4

*CRS* Composite reference standard

*P < 0.05;

### Xpert MTB/RIF assay for UTB detection

In this study, there were four types of tests in which all samples were involved, namely smear, Xpert MTB/RIF, culture, and TB-DNA, and the distribution of the test results and efficacy comparison are shown in Tables 2, 3. Compared with CRS, the Xpert test had a sensitivity of 78.9% (CI 0.094–0.650) and a specificity of 68.6% (CI 1.262–3.253). FPV was 14.3% (CI 0.232–0.684) and FNV was 42.3% (CI 1.325–8.005). In contrast, the sensitivity of the smear and culture tests was only 77.8% and 75%. Xpert MTB/RIF specificity and FPV were 68.6% and 14.3%, both higher than smear, culture, and TB-DNA. The McNemar test showed that the Xpert MTB/RIF test was significantly better than the smear and culture tests (78.9% vs. 77.8%, *P* < 0.05; 78.9% vs. 75%, *P* < 0.05).

**Table 2:** Comparative efficacy of AFB smear, Xpert, culture, and TB-DNA

Test	Sensitivity (CI)	Specificity (CI)	FPV(CI)	FNV(CI)
AFB	77.8% (7/9)*	33.6% (37/110)*	5.1% (2/39)*	33.6% (37/110)
	(0.128–2.691)	(0.941–1.149)	(0.083–0.323)	(7.986–12.451)
Xpert	78.9% (15/19)	68.6% (24/35)	14.3% (4/28)	42.3% (11/26)
	(0.094–0.650)	(1.262–3.253)	(0.232–0.684)	(1.325–8.005)
Culture	75.0% (3/4)*	27.6% (8/29)*	11.1% (1/9)*	87.5% (21/24)
	(0.106–7.477)	(0.771–1.339)	(0.525–1.775)	(0.183–6.665)
TB-DNA	83.3% (5/6)	38.5% (10/26)*	9.1% (1/11)*	76.2% (16/21)
	(0.051–2.876)	(0.881–1.616)	(0.462–1.181)	(0.361–14.737)

Sensitivity, specificity, false positivity, and false negativity values of AFB, Xpert, culture, and TB-DNA in the detection of urine samples from UTB participants

*AFB* Acid-Fast Bacilli smears *Xpert* Xpert MTB/RIF assay *culture* culture for MTB *TB-DNA Mycobacterium tuberculosis* deoxyribonucleic acid *CI* confidence interval *FPV* false positivity value *FNV* false negativity value

*P < 0.05

**Table 3:** Result of microbiological examination

Test	CRS positive		CRS negative		Total positive share in (%)
	Positive	Relative share in (%)	Positive	Relative Share in (%)	
AFB	7	8.8(7/80)	2	5.1 (2/39)	7.56 (9/119)**
Xpert	15	41.7(15/36)	4	14.3 (4/28)	29.69 (19/64)
culture	3	12.5(3/24)	1	11.1 (1/9)	12.12 (4/33)
TB-DNA	3	23.8(5/21)	1	9.1 (1/11)	18.75 (6/32)*

*CRS* composite reference standard *AFB* Acid-Fast Bacilli smears *Xpert* Xpert MTB/RIF assay *culture* culture for MTB *TB-DNA Mycobacterium tuberculosis* deoxyribonucleic acid

*P < 0.05

**P < 0.01

**Fig. 2:**
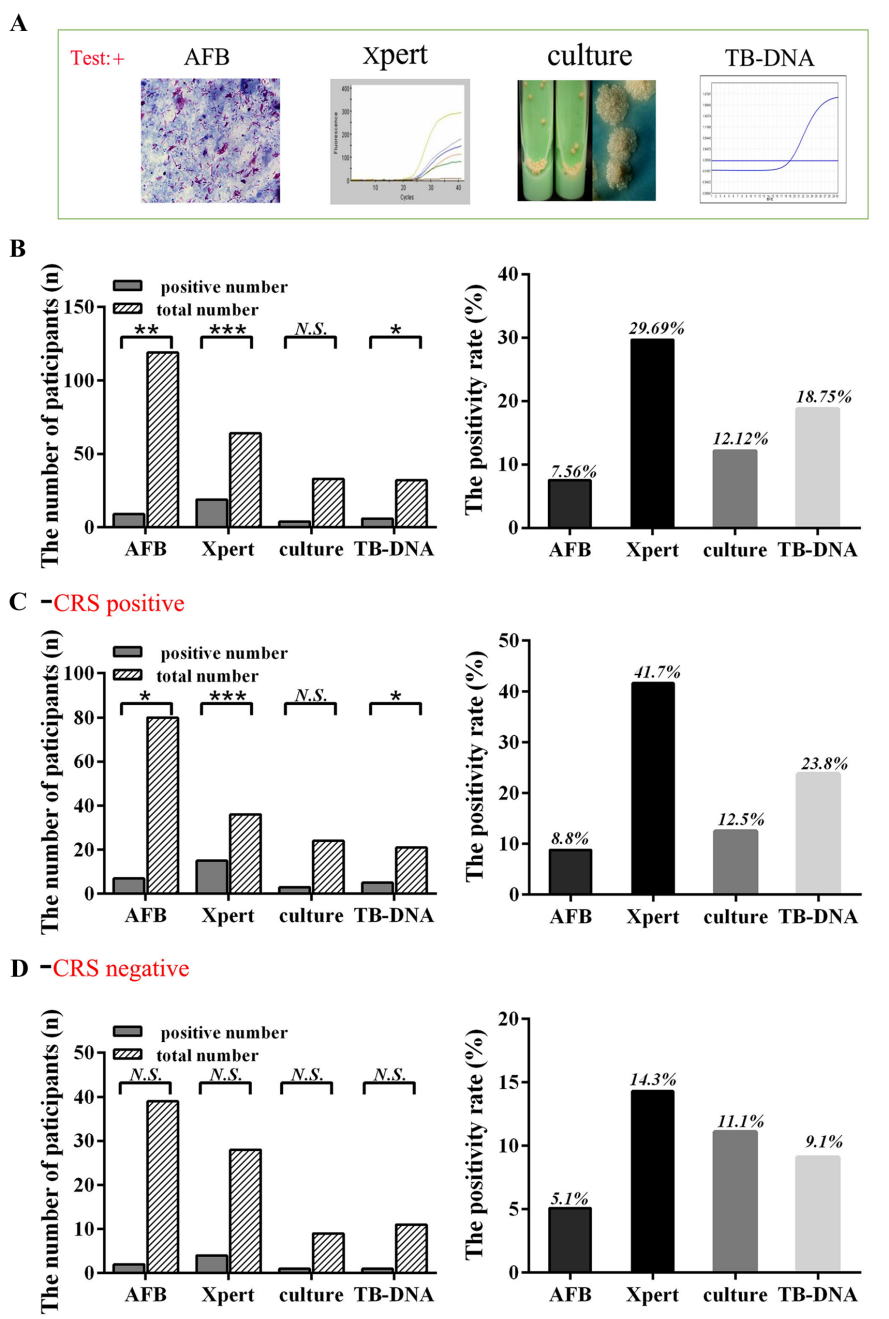
Results of microbiological examination. + positive *n* number *CRS* composite reference standard *AFB* Acid-Fast Bacilli smears *Xpert* Xpert MTB/RIF *culture* culture for MTB *TB-DNA Mycobacterium tuberculosis* deoxyribonucleic acid *N.S.* p > 0.05 *P < 0.1 **P < 0.01 ***P < 0.001

Using CRS as the gold standard, the total positivity rate of Xpert MTB/RIF was 29.69% (19/64, *P* < 0.001), far exceeding that of smear 7.56% (9/119, *P* < 0.01), culture 12.12% (4/33, *P* > 0.05), and TB-DNA 18.75% (6/32, *P* < 0.05), as shown in Fig. 2A, B.

Among the CRS-positive samples, the Xpert MTB/RIF positivity rate was 41.7% (15/36), higher than smears (8.8%), culture (12.5%), and TB-DNA (23.8%), as shown in Fig. 2C. Among CRS-negative samples, the positivity rate of Xpert MTB/RIF was 14.3% (4/28), which was higher than smear (5.1%), culture (11.1%), and TB-DNA (9.1%), as shown in Fig. 2A, D. As shown in Fig. 2, Xpert MTB/RIF had the highest positive detection rate in urine samples from all participants, both in CRS-positive and -negative participant samples.

### Xpert MTB/RIF has the highest positivity rate among smear negative

The low positivity rate of UTB smear affects the early diagnosis of UTB. Therefore, in this study, Xpert MTB/RIF, culture, and TB-DNA assays were performed for smear-negative samples, and analyzed their efficacy, as shown in Tables 4, 5. Under CRS, the sensitivity of the Xpert test was 75% (CI 0.086–1.605), and the specificity was 53.6% (CI 0.908–1.832) compared to smear negative. The FPV was 11.8% (CI 0.352–1.088) and the FNV 68.4% (CI 0.615–7.470). In contrast, the specificity of culture and TB-DNA examination was only 25% and 38.9%, and FPV was 0% for both. Compared to smear negatives, McNemar's assay showed that Xpert MTB/RIF assay specificity was significant for culture and TB-DNA (53.6% vs. 25%, *P* < 0.01; 53.6% vs. 38.9%, *P* < 0.05) and Xpert MTB/RIF assay FPV was significant for culture and TB-DNA (11.8% vs. 0%, *P* < 0.001; 11.8% vs. 0%, *P* < 0.001).

**Table 4:** Efficacy results for Xpert, MGIT 960 culture, and TB-DNA compared to AFB-negative results

Test	Sensitivity (CI)	Specificity (CI)	FPV(CI)	FNV(CI)
Xpert	75% (6/8)	53.6% (15/28)	11.8% (2/17)	68.4% (13/28)
	(0.086–1.605)	(0.908–1.832)	(0.352–1.088)	(0.615–7.470)
culture	100% (1/1)	25% (5/20)**	0% (0/5)***	75% (15/20)
	–	(0.940–1.211)	–	(0.582–0.966)
TB-DNA	100% (4/4)	38.9% (7/18)*	0% (0/7)***	61.1% (11/18)
	–	(1.005–1.850)	–	(0.432–0.883)

Sensitivity, specificity, false positivity, and false negativity values of AFB, Xpert, culture, and TB-DNA in the detection of smear-negative urine samples from UTB participants

*Xpert* Xpert MTB/RIF assay *culture* culture for MTB *TB-DNA Mycobacterium tuberculosis* deoxyribonucleic acid *CI* confidence interval *FPV* false positivity value *FNV* false negativity value

*P < 0.05

**P < 0.01

***P < 0.001

**Table 5:** Comparison with AFB-negative results

Tests	CRS + /AFB-	Relative share in (%)	CRS-/AFB-	Relative share in (%)	Total positive share in (%)
Xpert	6	31.6% (6/19)**	2	11.8% (2/17)	22.2 (8/36)*
Culture	1	6.2% (1/16)	0	− (0/5)	4.76 (1/21)
TB-DNA	4	26.7% (4/15)	0	− (0/7)	18.2 (4/22)

+ positive − negative *CRS* composite reference standard *AFB* Acid-Fast Bacilli smears *Xpert* Xpert MTB/RIF assay *culture* culture for MTB *TB-DNA Mycobacterium tuberculosis* deoxyribonucleic acid

*P < 0.05

**P < 0.01

**Fig. 3:**
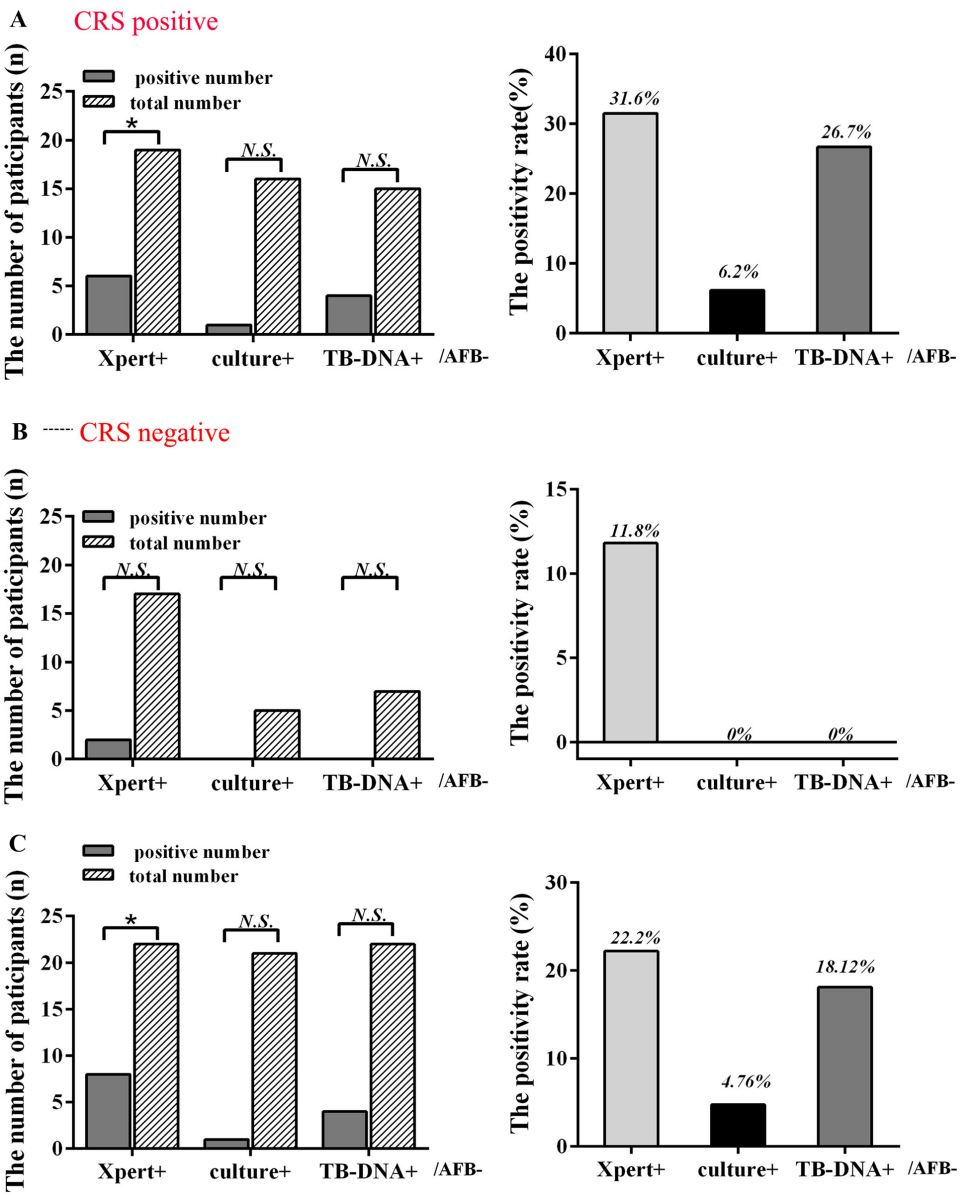
Comparison with smear-negative results. + positive − negative *n* number *CRS* composite reference standard *AFB* Acid-Fast Bacilli smears *Xpert* Xpert MTB/RIF *culture* culture for MTB *TB-DNA Mycobacterium tuberculosis* deoxyribonucleic acid *N.S.* p > 0.05 *P < 0.1 **P < 0.01 ***P < 0.001

Using CRS positive as the standard, in terms of smear-negative participants' urine samples, Xpert MTB/RIF positive detection rate is 31.6% (6/19, *P* < 0.1), far exceeding culture 6.2% (1/16, *P* > 0.05) and TB-DNA 26.7% (4/15, *P* > 0.05), as shown in Fig. 3A. Using CRS negative as the standard, in terms of smear-negative participants' urine samples, Xpert MTB/RIF positive detection rate is 11.8% (2/17, *P* > 0.05), however, neither culture nor TB-DNA tested positive, as shown in Fig. 3B. Therefore, for smear-negative urine samples, the positivity rate of Xpert MTB/RIF far exceeds that of culture and TB-DNA. Compared to smear negative, Xpert MTB/RIF, culture, and TB-DNA positive rates were 22.2% (8/36, *P* < 0.05), 4.76% (1/21, *P* > 0.05), and 18.2% (4/22, *P* > 0.05), respectively, as shown in Fig. 3C.

## Discussion

UTB is a common EPTB, and UTB is a part of systemic TB, most of which are secondary to TB. The typical symptoms are frequent urination, urgency, hematuria, or pyuria, and MTB testing is still needed for clinical diagnosis. Early diagnosis is very important to control the rapid spread of TB, but the detection of TB infection in the early stage is not easy. MTB culture is still the gold standard for clinical diagnosis of TB, but its cycle is long, the FNV and FPV are high, and the diagnosis of EPTB and smear-negative participants is very difficult [17]. Tuberculin pure protein derivative (PPD) experiments are simple, inexpensive, and widely used, but are affected by immune status and cross-infection, and have low sensitivity and specificity [18]. Conventional PCR to detect TB infection requires electrophoresis after amplification, which is fast but not quantitative, prone to cross-infection, and has low specificity. Real-time fluorescent quantitative PCR detection is an emerging method for diagnosing TB in recent years; it can quantitatively measure DNA and is widely used due to its fast speed and high accuracy. TB-DNA detection is fast and accurate and can be used as a rapid method for diagnosing TB. Still, it is difficult to identify when the non-specific amplification product is inconsistent with the target molecule. Due to the low positivity rate of traditional smears and the long time-consuming and complicated operation of MTB culture, a rapid and accurate diagnostic technique is needed in the clinic. Molecular diagnostic methods for TB have been promoted and used since the beginning of this century, and WHO issued policy guidance for Xpert MTB/RIF in 2011.

Studies in recent years have shown that Xpert MTB/RIF has high sensitivity and specificity for the diagnosis of pulmonary TB, but the sensitivity of different parts of EPTB is inconsistent [19]. The study found that the overall sensitivity of Xpert MTB/RIF to EPTB was 77%–78%, and the specificity was 97–99%. The sensitivity in pus and lymph fluid was up to 87%, and the sensitivity in effusion was lower. The sensitivity was 32% in pleural effusion and 29% in ascites [20, 21]. Penz et al. [22] conducted a meta-analysis of 36 studies on Xpert MTB/RIF detection in the diagnosis of EPTB, including 358 gastrointestinal (tissue mucosa) samples, with a sensitivity of 86% and a specificity of 98%. The results of Singh et al. showed that the sensitivity of Xpert MTB/RIF in detecting intestinal mucosal tissue was over 90%, and the specificity could reach 99.62%, while the detection of Xpert MTB/RIF in urine samples was rarely reported in the diagnosis of urinary TB [23]. In this study, compared with CRS, the sensitivity of Xpert MTB/RIF detection of urine was 78.9%, with 19 positive cases identified in 64 urine samples, which is close to the sensitivity of 69.09% previously reported by Samuel et al. [24]. The sensitivity of Xpert MTB/RIF identification in this study was lower than that of the previous study reported by Hillemann et al., i.e., 100.0%, but only 6 positive cases out of 91 urine samples in that study [25]. Lawn and Zumla [26] also evaluated 238 EPTB samples and reported a sensitivity of 87.5% (CI 71–100%) for the Xpert MTB/RIF assay in urine samples; however, only 6% of these 238 samples were urine samples. Another Italian study reported a sensitivity and specificity of 92.3 and 99.0%, respectively, with 15 positive cases (out of 130 urine samples) identified by Xpert MTB/RIF [13]. In contrast, our study had a higher statistical power. The differences may be due to the different urine samples used. However, as there were few strains with resistance in the available studies (5 strains in the study by Pang et al. [14] and 1 strain in our study), this requires further studies to validate the role of Xpert MTB/RIF in RIF drug resistance detection.

Compared with smear negative, the positivity rate of Xpert MTB/RIF was 22.2% (8/36, *P* < 0.05) significantly better than culture and TB-DNA, which is also generally consistent with that reported by Ciftci et al. [27]. In addition, the significant features of Xpert MTB/RIF detection technology are simple operation steps, short time consuming, MTB can be detected within 2 h, and RIF resistance can be judged at the same time, which is conducive to early diagnosis. Smear cannot detect granular and L-type MTB, nor can it distinguish NTM (non-tuberculous mycobacteria), and the false positivity rate further increases in the presence of increased mutant strains. However, Xpert MTB/RIF detects its specific DNA sequence and does not distinguish between live and dead bacteria, which improves the positivity rate of Xpert MTB/RIF. However, the positive Xpert MTB/RIF in participants cannot be used as the gold standard for the diagnosis of UTB, which may be related to some participants with severe PTB swallowing a part of sputum with MTB, but further research is needed. In addition to, Xpert MTB/RIF assay results for RIF resistance were sensitive in all participants, but the new generation of Xpert® MTB/RIF Ultra improves the sensitivity of MTB detection and further improves RIF resistance detection by increasing the volume and utilizing multi-copy amplification targets [28, 29]. It has been shown that Xpert® MTB/RIF Ultra outperforms Xpert MTB/RIF in diagnosing participants with smear-negative PTB [30]. Performance in extrapulmonary TB samples was also noted [31]. Xpert® MTB/RIF Ultra has been introduced in our laboratory, and questions about RIF resistance will be the focus of the next study.

## Conclusion

In conclusion, urine Xpert MTB/RIF detection can provide bacteriological evidence for UTB participants as soon as possible, so that they can receive timely and effective treatment, and prevent the occurrence of serious complications, such as bladder contracture, hydronephrosis, and spontaneous rupture of the tuberculous bladder.

## Data Availability

All data relevant to this study are included in the article and other raw datasets used for analysis during the current study are available from the corresponding author on reasonable request.

## Abbreviations

CRS: Composite reference standard
AFB: Acid-fast Bacilli smears
Gene Xpert MTB/RIF: Xpert® mycobacterium tuberculosis/rifampicin (MTB/RIF)
TB-DNA: Mycobacterium tuberculosis deoxyribonucleic acid
UTB: Urinary tuberculosis
RIF: Rifampicin
PTB: Pulmonary tuberculosis
CI: Confidence interval
FPV: False positivity value
FNV: False negativity value
LUTS: Lower urinary tract symptoms
IVU: Intravenous urography
CT: Computed tomography
